# A genetic polymorphism repurposes the G-protein coupled and membrane-associated estrogen receptor GPER to a transcription factor-like molecule promoting paracrine signaling between stroma and breast carcinoma cells

**DOI:** 10.18632/oncotarget.18156

**Published:** 2017-05-24

**Authors:** Marco Pupo, Alexandre Bodmer, Melissa Berto, Marcello Maggiolini, Pierre-Yves Dietrich, Didier Picard

**Affiliations:** ^1^ Département de Biologie Cellulaire and Institute of Genetics and Genomics of Geneva, Université de Genève, Sciences III, CH-1211 Genève 4, Switzerland; ^2^ Department of Pharmacy, Health and Nutritional Sciences, University of Calabria, Rende, Italy; ^3^ Département d’Oncologie, Hôpitaux Universitaires de Genève, CH - 1211 Genève 14, Switzerland; ^4^ Current address: Areta International S.r.l., Gerenzano, Italy

**Keywords:** breast cancer, cancer-associated fibroblasts, tumor microenvironment, nuclear localization, single nucleotide polymorphism

## Abstract

GPER is a membrane-associated estrogen receptor of the family of G-protein coupled receptors. For breast cancer, the contribution of GPER to promoting the proliferation and migration of both carcinoma cells and cancer-associated fibroblasts (CAFs) in response to estrogen and other agonists has extensively been investigated. Intriguingly, GPER was previously found to be localized to the nucleus in one isolate of breast CAFs. Moreover, this nuclear GPER was shown to bind regulatory sequences of cancer-relevant target genes and to induce their expression. We decided to find out what induces the nuclear localization of GPER, how general this phenomenon is, and what its functional significance is. We discovered that interfering with N-linked glycosylation of GPER, either by mutation of the predicted glycosylation sites or pharmacologically with tunicamycin, drives GPER into the nucleus. Surveying a small set of CAFs from breast cancer biopsies, we found that a relatively common single nucleotide polymorphism, which results in the expression of a GPER variant with the amino acid substitution P16L, is associated with the nuclear localization of GPER. GPER with P16L fails to be glycosylated, presumably because of a conformational effect on the nearby glycosylation sites. GPER P16L is defective for membrane-associated signaling, but instead acts like an estrogen-stimulated transcription factor. In CAFs, it induces the secretion of paracrine factors that promote the migration of carcinoma cells. This raises the possibility that the GPER P16L polymorphism could be a risk factor for breast cancer.

## INTRODUCTION

Breast cancer is the most frequently diagnosed type of cancer and the leading cause of cancer death among women worldwide [1]. The onset and the progression of breast tumors have been correlated with a wide variety of risk factors, including genetic predisposition and exposure to estrogens [2]. It is known that estrogens bind to specific nuclear receptors, the estrogen receptors α (ERα) and β, of which ERα generates a potent stimulus for the proliferation of breast epithelial cells and increases the risk of DNA mutation during replication [3]. A plethora of studies have demonstrated that ER-signaling functions as a major driver of breast cancer tumorigenesis, promoting cancer cell proliferation, survival, and invasive behavior [4]. However, some studies indicated that estrogens can promote breast cancer progression through ERα-independent mechanisms [5]. In particular, in the last few years, it has become increasingly evident that the unrelated G protein-coupled estrogen receptor (GPER, formerly also known as GPR30) mediates some of the non-ERα signaling stimulated by estrogens [6]. GPER has been shown to mediate effects triggered by estrogens, antiestrogens and xenoestrogens, including rapid MAPK activation, the induction of early gene expression, proliferation and migration in different types of normal and malignant cell types [7–12]. The signal transduction mechanism of GPER has therefore extensively been studied. Intriguingly, the intracellular localization of the receptor has remained controversial. Although GPER belongs to a cell surface receptor family, which conventionally mediates transmembrane signaling of membrane-permeable as well as membrane-impermeable ligands, numerous studies demonstrated that GPER is detectable not only at the plasma membrane, but also at intracellular membranes [13–15]. Moreover, recent studies showed a peculiar GPER localization in the nucleus of breast cancer-associated fibroblasts (CAFs); this nuclear GPER was even found to be recruited in an estrogen-stimulated fashion to chromatin at target genes such as *c-FOS* and *CTGF* leading to increased expression of these genes [16, 17].

The tumor stroma, of which CAFs are an important component, represents a driving force to sustain cancer progression. In the last few years, it has become clear that there is a reciprocal interplay between tumor cells and the microenvironment. It has been demonstrated that this close relationship is involved in promoting the progression of neoplasms through the stimulation of invasion, angiogenesis and metastasis [18, 19]. Hence, the components of the tumor microenvironment have received growing attention in order to understand the molecular signaling pathways that are active in these cells. The tumor microenvironment is composed of cellular and non-cellular components. Multiple different cell types comprise the cellular compartment of the tumor microenvironment, including immune cells, endothelial cells and fibroblasts. Fibroblasts are the most abundant cell type in the tumor-associated stroma, with multiple roles, including deposition of extracellular matrix and basement membrane components, regulation of differentiation events in associated epithelial cells, modulation of immune responses and maintenance of homeostasis [20]. Several studies have highlighted the important role of GPER in mediating estrogen signaling in CAFs and, in particular, its contribution to paracrine signaling between stroma and cancer cells [21–23].

However, the importance of the unusual presence of GPER in the nucleus of breast CAFs as well as the determinants that underlie the nuclear accumulation of the receptor are unclear. Recently, we demonstrated that the nuclear localization of GPER in CAFs is importin-dependent and that a nuclear localization signal is present within the GPER protein sequence [16]. The G-protein coupled receptors (GPCRs), such as GPER, were long believed to trigger biological responses by binding to their external ligands exclusively at the cell surface [24]. This model has been challenged in recent years by the discovery of functional intracellular GPCRs in different cellular compartments, including the nucleus. So far, more than 30 different GPCRs have been detected in nuclei of different tissues and in different cellular contexts [25, 26]. Hence, the plasma membrane can no longer be considered the exclusive signaling locus of GPCRs. In contrast, little is known about how GPCRs are targeted to the nucleus. Several studies showed the presence of heptahelical receptors in the nucleus in a constitutive manner, suggesting that their nuclear translocation is not dependent on the binding of their cognate ligands [27]. These findings suggest that GPCRs may be trafficking directly to the inner nuclear membrane after biosynthesis and assembly in the endoplasmic reticulum. Uncharacterized events following synthesis may determine their final destination. In this context it is worth noting that several studies demonstrated that the elimination of N-glycosylation sites in certain GPCRs can lead to their accumulation in the nuclear and perinuclear compartments [28, 29]. Whether and how GPCRs may even be soluble within the nucleus also remains enigmatic.

We therefore decided to investigate what determines the nuclear localization of GPER and to explore the functional significance of this phenomenon in CAFs from breast cancers. Our data provide novel insights into the role of nuclear GPER in CAFs, further highlighting the importance of estrogenic signals acting through GPER in the stroma for promoting breast cancer progression.

## RESULTS

### Non-glycosylated GPER accumulates in the nucleus

**Figure 1:**
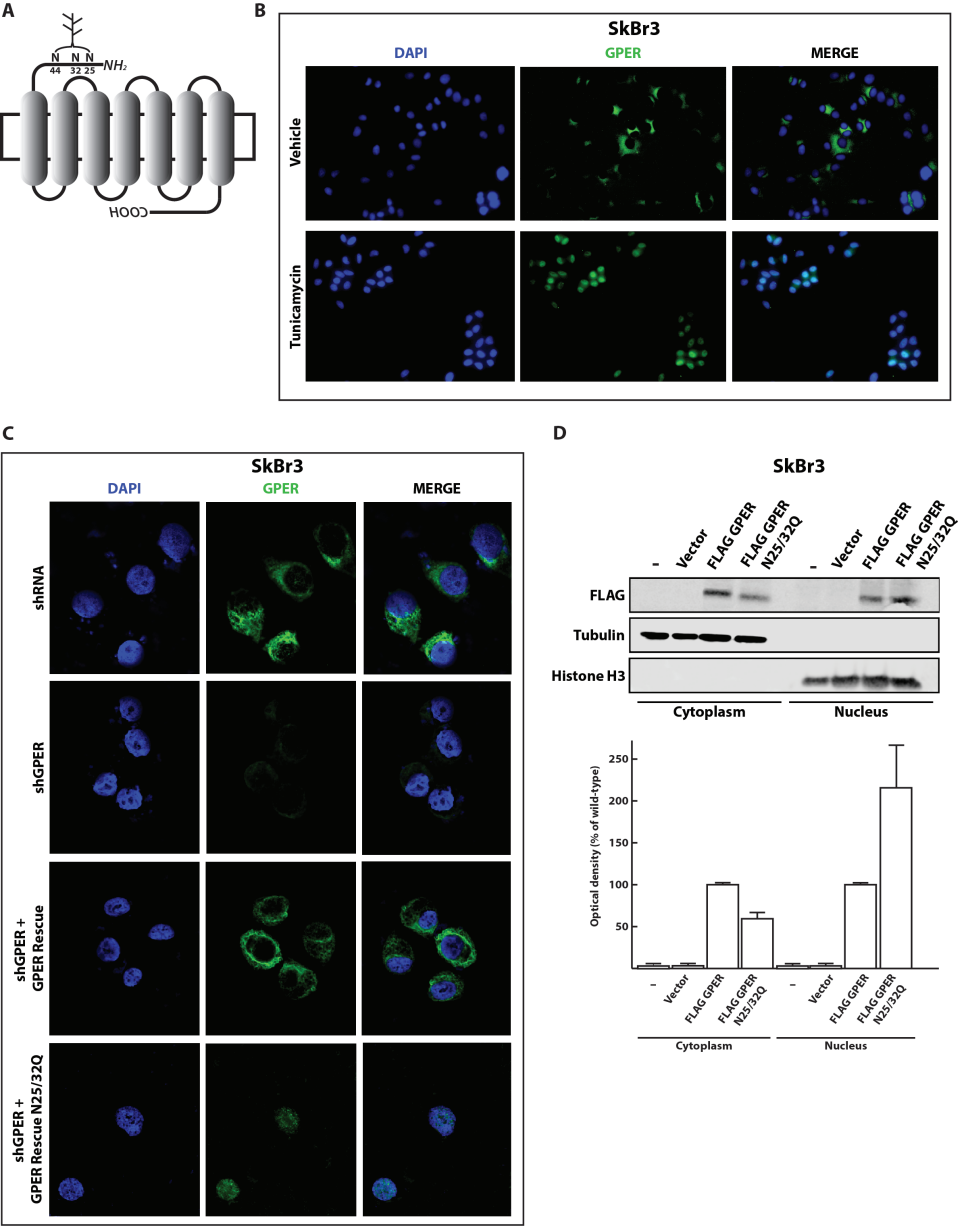
Inhibition of N-linked glycosylation induces the nuclear accumulation of GPER **A**. Schematic representation of the GPER protein structure. The asparagine residues N25, N32 and N44 are predicted N-linked glycosylation sites in the N-terminal portion of the receptor. **B**. Representative immunofluorescence micrographs of endogenous GPER in SkBr3 breast cancer cells treated or not for 24 hours with 5 μg/ml of tunicamycin and stained with an anti-GPER antibody (green staining). Nuclei were stained with DAPI (blue). Images shown are representative of 10 different random fields. **C**. GPER immunoflourescence micrographs of SkBr3 cells transfected for 24 hours with shRNA or shGPER constructs, or co-transfected, for additional 24 hours, with shGPER in combination with the shRNA-resistant GPER expression vector for either the wild-type GPER (GPER Rescue) or the N-glycosylation double mutant N25/32Q. Images shown are representative of 10 different random fields. **D**. Immunoblot of a subcellular fractionation experiment with SkBr3 breast cancer cells transfected as indicated. For these experiments, exogenous GPER is FLAG-tagged. Tubulin and histone H3 serve as markers and for standardization for the cytoplasmic and nuclear compartments, respectively. The bottom panel shows the densitometric analysis with each data point representing the mean ± SD of three independent experiments. After standardization to the respective compartmental markers, the values were expressed as % of the respective samples with FLAG-tagged wild-type GPER (each set to 100%).

Previous studies had already correlated the lack of glycosylation on asparagine with non-canonical localization of GPCRs in different experimental models [28–31]. Therefore, we initially aimed to determine whether changes in the N-linked glycosylation status of GPER could be associated with its nuclear localization in breast CAFs. We used the online tool NetNGlyc (http://www.cbs.dtu.dk/services/NetNGlyc) to scan the GPER protein sequence for putative N-linked glycosylation sites. In agreement with previous observations [32], the three asparagine residues 25, 32 and 44 in the N-terminal and presumably luminal portion of GPER could be N-linked glycosylation sites (Figure 1A and Supplemental Figure 1A). To determine whether the glycosylation status of GPER correlates with its intracellular localization, we treated SkBr3 breast cancer cells, which express endogenous GPER, with 5 μg/ml tunicamycin for 24 h. This drug treatment should prevent glycosylation. As shown in the immunofluorescence micrographs of Figure 1B, in cells treated with tunicamycin, GPER clearly accumulates in the nucleus suggesting that glycosylation is important for its cytoplasmic retention. To further corroborate our observations, we used site-directed mutagenesis to mutate specifically two out of the three predicted N-glycosylation sites. Using an shRNA-resistant version of *GPER1* as a wild-type backbone, we changed the codons encoding the asparagine residues 25 and 32 to encode glutamine. Then, we transfected SkBr3 cells with the plasmids for wild-type GPER or for the double mutant N25/32Q in combination with an shRNA construct against the endogenous GPER (shGPER). As expected, the endogenous and cytoplasmically localized GPER disappeared in cells transfected with shGPER (Figure 1C). Upon transfection of the SkBr3 cells with both shGPER and the wild-type GPER expression vector, the receptor was localized in the cytoplasm as determined by immunofluorescence. In contrast, the GPER double mutant N25/32Q tested under the same conditions clearly accumulated in the nucleus (Figure 1C).

To confirm these data biochemically, we performed cell fractionation followed by immunoblotting experiments after transfecting SkBr3 cells with a FLAG-tagged wild-type or mutant GPER. As shown in Figure 1D, the proportion of nuclear *versus* cytosolic GPER was increased for the double mutant compared to the wild-type protein. In addition, the immunoblotting experiments indicated a slight size difference between the wild-type and mutant proteins. Upon digesting the protein lysates with the enzyme EndoH to remove the N-linked glycosyl moieties, this difference was no longer evident suggesting that the size difference is due to a different glycosylation status of the proteins (Supplemental Figure 1B). Overall, these data demonstrate that glycosylation is required for the cytoplasmic localization of GPER and that interfering with its glycosylation induces its accumulation in the nucleus.

### A CAF isolate with nuclear GPER contains a single nucleotide polymorphism in *GPER*

**Figure 2:**
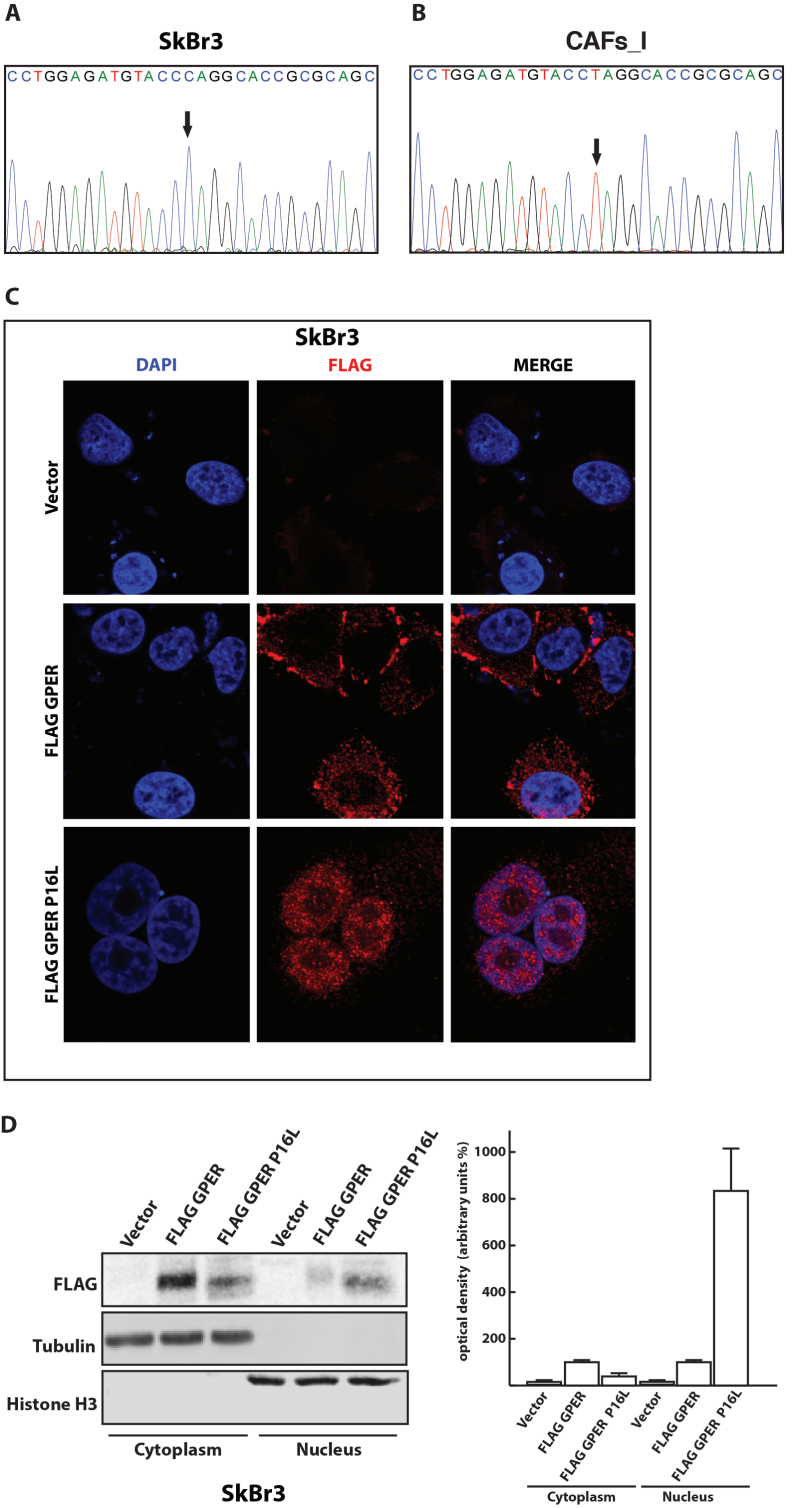
The P16L polymorphism of GPER is present in CAFs_I and promotes the nuclear localization of GPER in transfected SkBr3 cells Direct amplicon sequencing of the *GPER1* portion encoding the N-terminus of GPER of SkBr3 breast cancer cells **A**. and CAFs_I **B**. Note that CAFs_I are homozygous for the variant T allele that leads to the P16L substitution in the protein. **C**. Subcellular localization of FLAG-tagged wild-type and P16L variant GPER in transfected SkBr3 cells. The immunofluorescence images shown are representative of 10 different random fields. **D**. Immunoblot of a subcellular fractionation experiment with SkBr3 breast cancer cells transfected as indicated. Exogenous GPER is FLAG-tagged, endogenous tubulin and histone H3 serve as markers and for standardization for the cytoplasmic and nuclear compartments, respectively. The panel on the right shows the result of the densitometric analysis with each data point representing the mean ± SD of three independent experiments. After standardization to the respective compartmental markers, the values were expressed as % of the respective samples with FLAG-tagged wild-type GPER (each set to 100%).

To determine whether the nuclear localization of GPER that we had previously observed in a particular isolate of CAFs (here, referred to as CAFs_I) [16, 17] could be due to the presence of a mutation in its N-terminal domain, we sequenced the relevant genomic region of the *GPER1* gene. As shown in Figure 2A and 2B, we found a homozygous C to T mutation in CAFs_I relative to SkBr3 cells. This mutation results in a proline to leucine substitution at position 16 of the GPER protein sequence. Interestingly, this corresponds to the single nucleotide polymorphism (SNP) rs11544331, which had previously been highlighted in the *GPER1* sequence (http://www.ncbi.nlm.nih.gov/SNP/snp_ref.cgi?rs = 11544331; see Discussion). To test directly whether this SNP has an impact on the glycosylation and nuclear localization of GPER, we constructed a plasmid to express the FLAG-tagged GPER mutant P16L. We expressed this mutant in SkBr3 breast cancer cells and performed both immunofluorescence and cellular fractionation experiments. As shown in Figure 2C and 2D, the P16L mutant clearly accumulated in the nucleus suggesting that the mutation P16L promotes the nuclear localization. The punctate pattern of GPER staining both in the cytoplasm and in the nucleus (Figure 2C) is not an artefact of the FLAG-tagged version of GPER or of the anti-FLAG antibody used in this particular experiment. We do not know what the significance of this pattern is and what other cellular structure it corresponds to, but it is also commonly seen with antibodies to endogenous GPER (see Figure 1 and below, and ref. 16). The altered subcellular distribution of the P16L mutant is further supported by the biochemical experiments and a size shift compatible with an impact on the glycosylation status (Figure 2D). Overall, these data demonstrate that it is the P16L polymorphism present in CAFs_I that somehow affects the N-glycosylation status of GPER and promotes its nuclear localization.

### An expanded set of breast CAFs contains the P16L polymorphism

In order to extend the cohort of patients beyond the original singular CAFs_I, we obtained CAFs from biopsies of 5 invasive breast tumors (patients #1 to #5) and of 3 in-situ carcinomas (patients #6 to #8). Moreover, for all patients used in this study we obtained a piece of normal tissue from the same breast. CAFs and normal fibroblasts extracted from the aforementioned biopsies were checked for the expression of the mesenchymal marker vimentin and the epithelial marker E-cadherin. All cells obtained from breast cancer specimens and normal tissue, as expected, expressed only vimentin (Supplemental Figure 2A-2B and Supplemental Figure 3A-3B). Given the lack of staining with the antibody against E-cadherin, we used epithelial cells from another breast cancer biopsy as a positive control (Supplemental Figure 3C). To confirm that fibroblast-like cells isolated from breast cancer specimens were really CAFs, we used quantitative RT-PCR to evaluate the relative expression of three different markers of activation. Specifically, we selected the mRNAs for Fibroblast Activation Protein (*FAP*), smooth muscle actin (*ACTA2*) and caveolin-1 (*CAV1*). The expression levels of *FAP* and *ACTA2* in putative CAFs from breast tumors were higher than in fibroblasts from normal tissue (Supplemental Figure 4A-4B), whereas *CAV1* expression was lower in putative CAFs from breast carcinoma samples than in fibroblasts from normal tissues (Supplemental Figure 4C). Previous studies had demonstrated that the original CAFs_I used here only express GPER and are ERα-negative [16–17]. We could confirm this pattern by RT-PCR and immunoblotting experiments for all fibroblast samples from our biopsies (Supplemental Figure 5A-5H).

**Figure 3:**
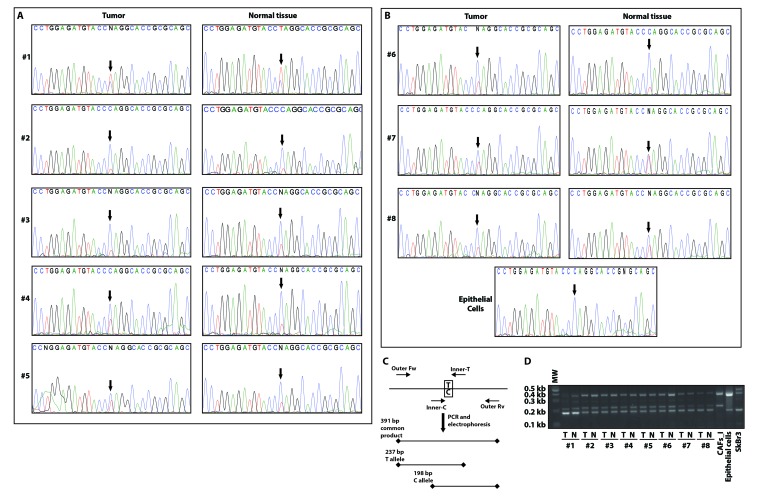
CAFs and normal fibroblasts from a panel of breast cancer biopsies are all heterozygous for the P16L polymorphism Direct amplicon sequencing of the *GPER1* portion encoding the N-terminus of GPER of CAFs and normal fibroblasts from patients #1-5 with invasive carcinoma **A**. and from patients #6-8 with in-situ carcinoma and independent normal epithelial cells for comparison **B**. Note that all fibroblasts in this set of samples display a double peak with both C and T at the relevant position (arrows). **C**. Scheme of the analytical PCR protocol to confirm the presence of the C/T polymorphism indicated by sequencing. Using two common outer primers (Outer Fw and Outer Rv) and two allele-specific inner primers (Inner-C and Inner-T), PCR products of specific diagnostic sizes are generated. **D**. Image of an agarose gel for the genotyping of all samples with the PCR scheme of panel C. T and N stand for tumor (CAFs) and normal fibroblasts, respectively. Note that fibroblasts of patients #1-8 are heterozygous, CAFs_I homozygous for the variant with the T allele, and eptihelial cells from an unrelated breast carcinoma sample and SkBr3 cells are homozygous for the C allele.

We next explored the presence of the P16L polymorphism in our new set of CAFs by PCR amplification and direct sequencing of an appropriate *GPER1* amplicon. Surprisingly, all samples proved to be heterozygous for the C/T polymorphism causing the P16L substitution, both those from the tumors themselves and those from the corresponding normal tissues (Figure 3A-3B). To confirm these data by an independent approach, we used a single-tube allele-specific PCR protocol [33] (Figure 3C). Again, we observed the presence of both alleles in all samples of CAFs and their corresponding normal fibroblasts (Figure 3D). Applying this PCR protocol to the control samples (CAFs_I, unrelated breast cancer epithelial cells and SkBr3 cells) confirmed their homozygosity for their respective alleles. The T of the T allele lying within the sequence CCTAGG, the restriction enzyme AvrII can be used to probe for its presence. The digestion pattern of the *GPER1* amplicon from some representative samples fully confirmed the data obtained with the other assays (Supplemental Figure 6A-6B). Overall, these data, obtained using three different techniques, demonstrated that the C/T substitution, responsible for the P16L polymorphism, is present in both alleles of the original CAFs and in one allele of all new biopsies.

### The polymorphic GPER localizes to both the cytoplasm and the nucleus in fibroblasts breast cancer biopsies

**Figure 4:**
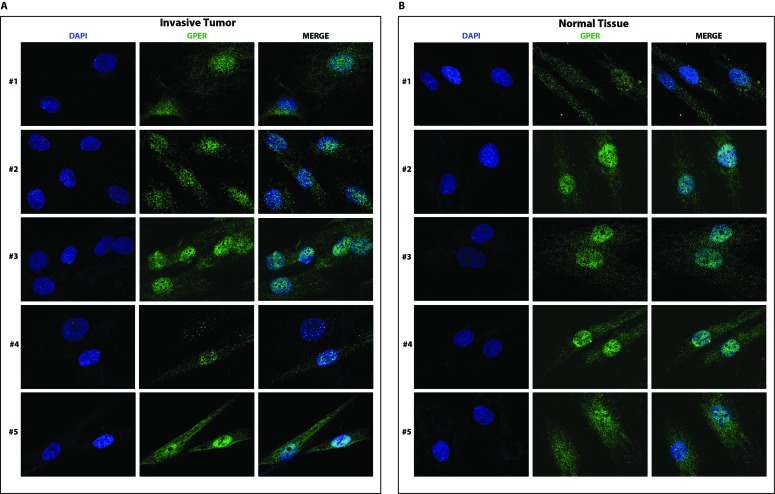
GPER localizes to both in the cytoplasm and the nucleus of CAFs A. and normal fibroblasts B. from invasive breast cancer biopsies Samples are from patients #1-5 with invasive breast carcinoma. Samples were stained with an anti-GPER antibody (green) and DAPI (blue), and analyzed by confocal microscopy. Immunofluorescence micrographs are representative of 10 different random fields.

**Figure 5:**
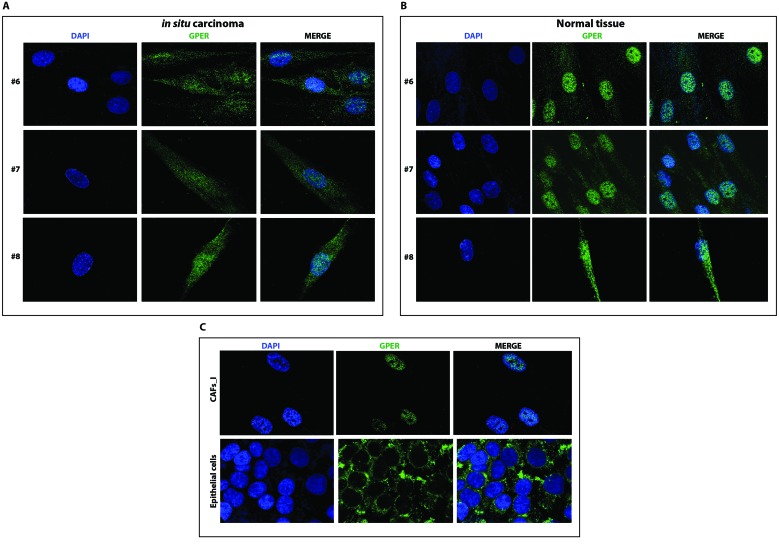
GPER localizes to both in the cytoplasm and the nucleus of CAFs A. and normal fibroblasts B. from biopsies of in-situ breast carcinoma Samples are from patients #6-8. **C**. Immunofluorescent staining of CAFs_I and epithelial cells from an unrelated breast carcinoma sample. Samples were stained with an anti-GPER antibody (green) and DAPI (blue), and analyzed by confocal microscopy. Immunofluorescence micrographs are representative of 10 different random fields.

Having established that our new set of CAFs from neoplastic breast samples are heterozygous for both GPER alleles, we determined the subcellular localization of GPER in these samples using a specific anti-GPER antibody (Supplemental Figure 7). As shown in Figure 4A and 4B and in Figure 5A and 5B, GPER localizes to both the nucleus and the cytoplasm in all samples of fibroblasts from breast cancer biopsies (both invasive and in-situ) as well as in the fibroblasts from the corresponding normal tissues. In the original CAFs_I, which are homozygous for the T allele, and in the unrelated epithelial cells, which are homozygous for the C allele, GPER is clearly only localized in the nuclear and cytoplasmic compartments, respectively. Thus, the specific GPER allele dictates the subcellular localization of GPER, and the P16L subset of GPER molecules account for those localized in the nucleus in cells from our new set of breast cancer biopsies.

### Nuclear GPER binds to regulatory sequences of target genes

**Figure 6:**
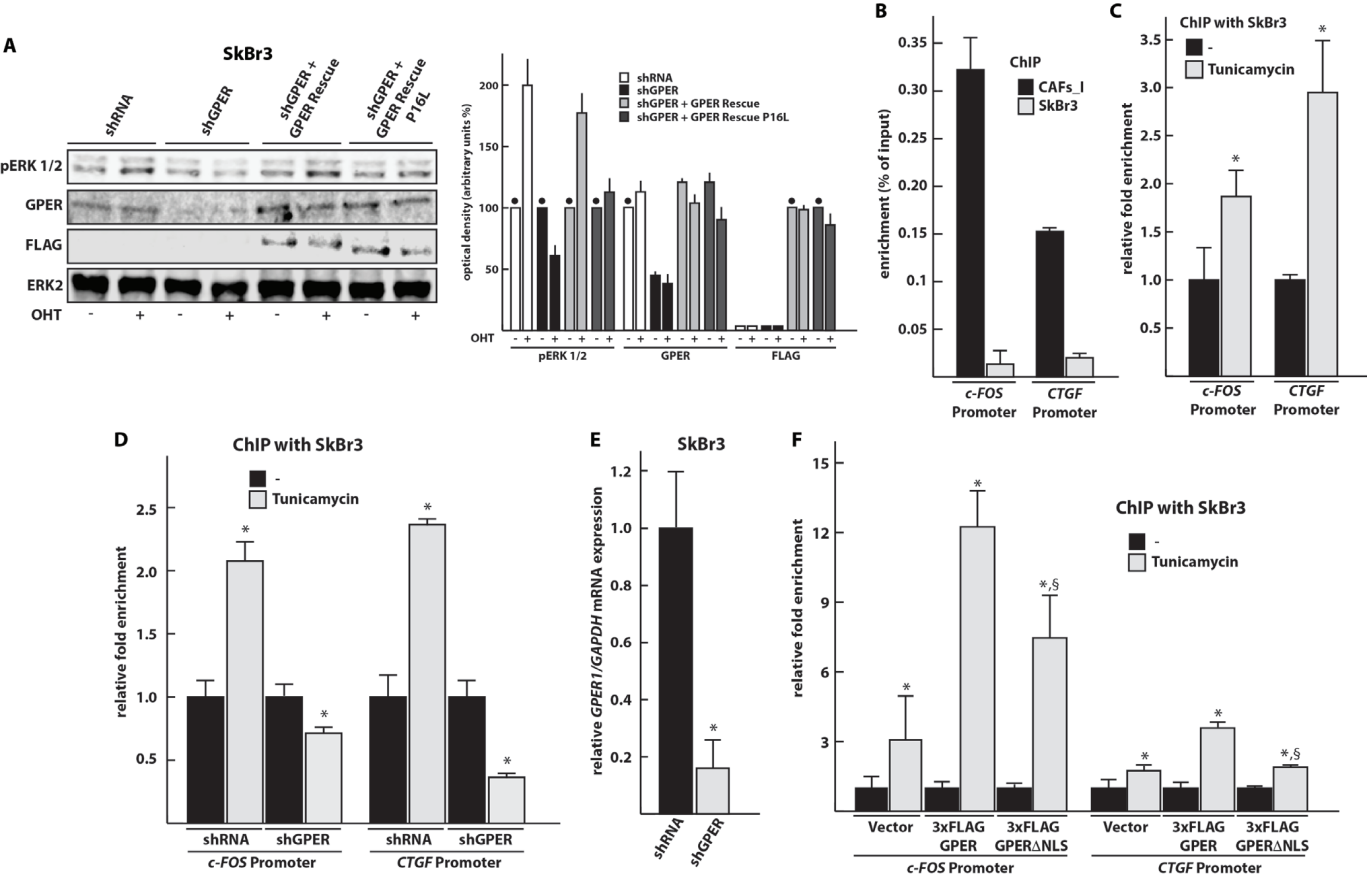
GPER localized to the nucleus is unable to stimulate MAPK signaling, but can be recruited to the promoters of its target genes *c-FOS* and *CTGF* **A**. Immunoblots showing ERK1/2 phosphorylation upon activation of GPER with OHT. Other panels display the total levels of ERK2, and the levels of endogenous GPER and exogenous FLAG-GPER in transfected SkBr3 cells. The transfected SkBr3 cells were treated for 30 min with vehicle (-) or 10 μM OHT. The panel on the right shows the densitometric quantitation of the blots normalized to ERK2 expression levels; each bar represents the average of two independent experiments with the lines on top indicating the range of the two values; the black dots indicate the samples, arbitrarily set to 100%, relative to which the values of the other samples of a set were calculated. **B**. GPER is recruited to the promoters of its target genes *c-FOS* and *CTGF* in CAFs_I. CAFs_I and SkBr3 cells were used to perform a ChIP assay with an anti-GPER antibody. **C**. Tunicamycin treatment induces the recruitment of GPER on the promoters of its target genes in SkBr3 cells, treated for 24 hours with 5 μg/ml tunicamycin and then used to perform ChIP experiments using an anti-GPER antibody. **D**. ChIP experiment to assess recruitment of GPER to target sites with SkBr3 cells transfected for 24 hours with shRNA or shGPER constructs and then treated for additional 24 hours with 5 μg/ml tunicamycin. ChIP experiments were performed using an anti-GPER antibody. **E**. Control RT-PCR experiment to verify the effectiveness of shGPER-mediated knock-down of the *GPER* mRNA 48 hours after transfection. Values were normalized to *GAPDH* expression, and presented as fold change (mean ± SD) of shGPER transfected cells relative to cells transfected with a control shRNA construct. **F**. ChIP experiment to assess recruitment of NLS-defective GPER to target sites. In this case, the ChIP was done with an anti-FLAG antibody. In ChIP experiments, the specific precipitation of AT-rich sequences from the *c-FOS* and *CTGF* promoters was evaluated by real-time PCR; enrichment was calculated relative to input (in panel B) as well as standardized to the untreated controls set to 1 (in panels C, D, and F). Bar graphs show the means ± SD of three independent experiments. *, p-value ≤ 0.05 for comparison to respective untreated (or shRNA) control; §, p-value ≤ 0.05 for comparison to corresponding wild-type GPER plasmid (panel F).

Previous studies had shown that ligand-activated GPER is able to induce MAPK signaling as indicated by ERK1/2 phosphorylation in different cellular contexts [9, 10, 34]. However, recently it was demonstrated that the polymorphic P16L GPER is unable to do so in vascular smooth muscle cells [35]. Therefore, we wanted to evaluate this in our model system. We co-transfected SkBr3 cells with the shGPER construct together with the expression vector for either wild-type GPER or the P16L variant. As shown in Figure 6A, the potent GPER agonist hydroxytamoxifen (OHT) is no longer able to induce the phosphorylation of ERK1/2 in cells co-transfected with shGPER and GPER P16L whereas this is clearly seen with wild-type GPER.

Since P16L GPER is defective for the activation of ERK1/2, we decided to find out whether it can still be recruited to the promoters of its two target genes *c-FOS* and *CTGF*. Having demonstrated that P16L GPER of CAFs accumulates in the nucleus, we used chromatin immunoprecipitation (ChIP) experiments to determine that and found, as expected [16], that GPER is able to bind the regulatory sequences of *c-FOS* and *CTGF* in CAFs_I (Figure 6B). In contrast, the wild-type and cytoplasmic version of GPER present in SkBr3 cells is poorly recruited to these chromatin sites unless its nuclear localization is triggered with the N-glycosylation inhibitor tunicamycin (Figure 6C). Binding is dependent on the presence of GPER since the signal disappears upon knocking it down with shGPER (Figure 6D and 6E). As an additional negative control, we exploited our previous finding that the nuclear localization of GPER depends on a cryptic C-terminally located nuclear localization signal (NLS) [16]. We expressed an NLS mutant version of GPER in SkBr3 cells. The chromatin recruitment of this version of GPER, which fails to accumulate in the nucleus, even in the presence of tunicamycin, is strongly reduced (Figure 6F).

### Overexpression of polymorphic nuclear GPER in CAFs stimulates the migration of breast cancer cells through paracrine signaling

**Figure 7:**
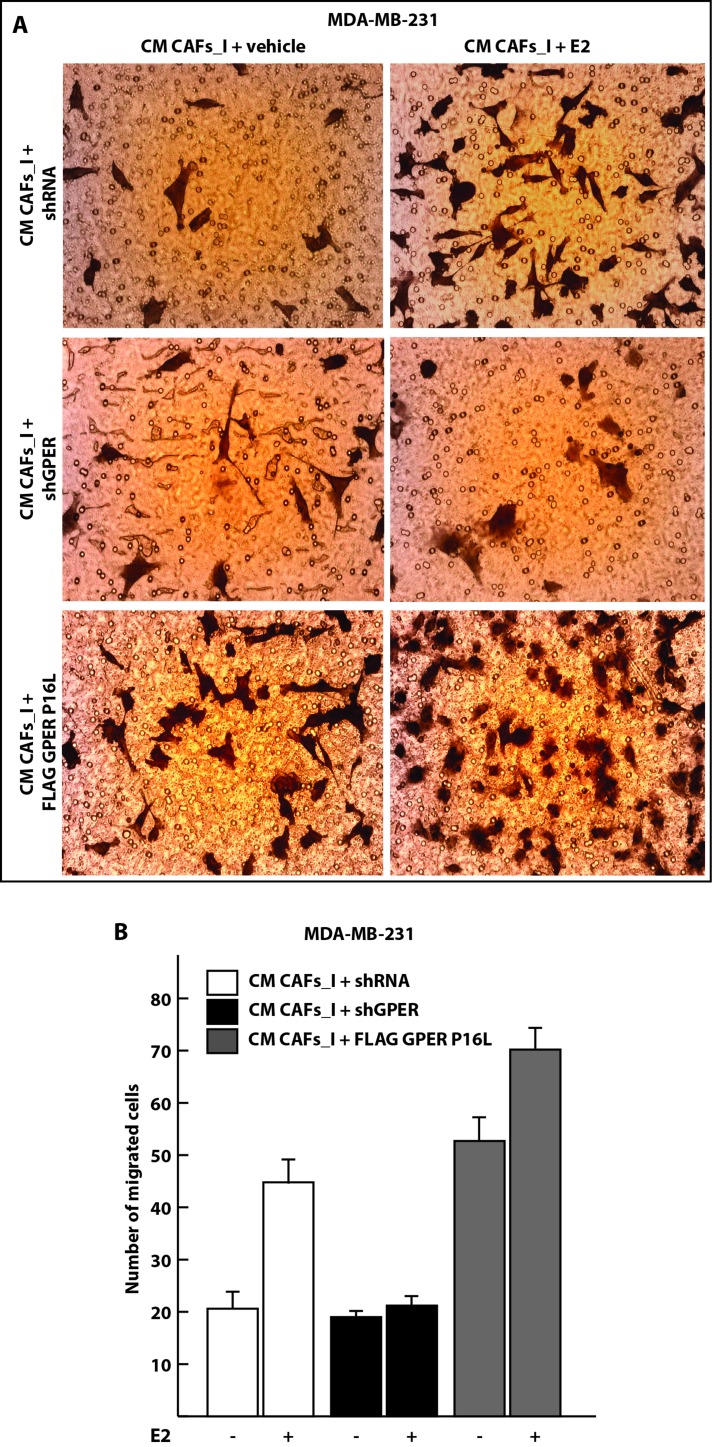
Conditioned medium from CAFs overexpressing the nuclear GPER P16L variant induces the migration of MDA-MB-231 breast cancer cells **A**. Representative micrographs of Boyden chamber assays performed with MDA-MB-231 cells treated with conditioned medium (CM) from CAFs. CAFs_I were transfected for 24 hours with control shRNA or shGPER constructs or a P16L mutant version of the GPER expression vector (FLAG GPER P16L) and then treated for 18 hours with 100 nM E2 where indicated. **B**. Quantitation of the Boyden chamber assays shown in panel **A**. Values represent the mean ± SD of the number of migrated cells counted in 10 random fields. Data are representative of two independent experiments performed with triplicate samples.

To explore the biological functions of GPER in the nucleus of CAFs, beyond the regulation of some target genes, we evaluated its impact on the migration of neighboring carcinoma cells. To this end, we overexpressed the wild-type and P16L versions of GPER in CAFs_I, treated them with 17-β-estradiol (E2), collected conditioned medium (CM) from them and used it in migration assays with MDA-MB-231 breast carcinoma cells. The migration of MDA-MB-231 cells was stimulated in the presence of CM from CAFs_I treated with E2. In contrast, the CM of CAFs_I transfected with shGPER was no longer able to induce the migration of MDA-MB-231cells, irrespective of the treatment with E2 (Figure 7A-7B). Most importantly, when we used the CM from CAFs_I cells overexpressing the P16L variant, migration of the breast cancer cells was strongly up-regulated, and it was further boosted when the CM was from CAFs_I cells that had been treated with E2 (Figure 7A-7B). Thus, nuclear GPER in CAFs appears to stimulate the expression and secretion of paracrine factors that induce the migration of adjacent breast carcinoma cells.

## DISCUSSION

The intracellular mechanisms, which regulate the behavior of the cellular components of the tumor microenvironment, have received growing attention. The role of CAFs in promoting cancer progression has been extensively studied [36]. The functions of GPER in mediating estrogenic signaling as well as drug resistance in CAFs have recently been highlighted [21, 37]. However, since the first intriguing observation that GPER localizes in the nucleus of CAFs from one particular breast cancer patient [17], the functional significance of this particular subcellular localization has remained rather enigmatic. It did raise the possibility that the alternate subcellular localization of GPER in CAFs and potentially in the carcinoma cells themselves may change the behavior of these cells and affect tumor progression.

Our present work demonstrates that it is the lack of N-linked glycosylation of GPER, which drives its nuclear localization. Glycosylation of GPER can be disrupted by a genetic polymorphism or pharmacologically. The latter suggests that there might even be physiological or pathological conditions that impair the glycosylation of GPER and promote its nuclear localization. In any case, this unusual subcellular localization seems to have functional consequences; we discovered that CAFs with nuclear GPER produce secreted factors that fuel the migration of nearby breast cancer cells. In light of our recent finding that GPER is at the heart of a positive feedforward loop between CAFs and breast carcinoma cells mediated by interleukin 1β (IL1) [21], it is likely that IL1β is at least one of these putative paracrine factors. Since CAFs secrete many other factors [38], a systematic screen will be necessary to determine which ones are relevant. Considering this regulatory interaction between stromal and carcinoma cells, it may be worth thinking about specifically interfering with the nuclear functions of GPER as a novel therapeutic strategy.

### Impairment of N-glycosylation promotes the nuclear localization of GPER

Nuclear localization has been reported for numerous other GPCRs [25], but the molecular mechanisms remain poorly understood. Even though lateral diffusion from the membrane of the endoplasmic reticulum through the nuclear pores is suggested to be the primary mechanism of localization for some resident nuclear membrane proteins like the lamin B receptor [39], it is unlikely to be the major pathway for the translocation of GPCRs that require further maturation of N-linked glycosyl modifications in the trans-Golgi network [40]. Unlike GPCRs such as the coagulation factor II receptor-like 1 (F2rl1) and the oxytocin receptor, which translocate to the nucleus following agonist stimulation at the cell surface [41, 42], GPER can be localized in the nucleus in the absence of agonist [16] and in an endocytosis-independent manner (data not shown). Whatever the underlying molecular mechanism is, impairing the N-glycosylation of GPER triggers its nuclear localization. This is reminiscent of studies on other GPCRs that correlated the lack of glycosylation with an altered intracellular localization including an accumulation near the nuclear compartment [28, 29]. We had previously demonstrated that the nuclear localization of GPER depends on a cryptic C-terminally located NLS [16]. Hence, N-glycosylation can be viewed as a mechanism to restrain GPER to the membranes of the endoplasmic reticulum and the cell surface.

### GPER P16L variant is not glycosylated and accumulates in the nucleus

The T allele of the SNP rs11544331 in the *GPER1* gene results in the expression of the P16L variant of GPER. The P16L variant fails to be glycosylated, presumably because it locally perturbs the conformation of the protein loop containing the glycosylated asparagine residues nearby. As a consequence, P16L localizes to the nucleus even in cells where GPER is normally localized outside of the nucleus. SNPs that affect the glycosylation status of a protein directly or indirectly have been reported before. For instance, a common SNP in the μ-opioid receptor gene alters an N-glycosylation site of the receptor and a SNP in the human serotonin transporter gene introduces a new site for N-linked glycosylation [43, 44]. An indirect effect similar to the impact of P16L in GPER was recently reported for CD23, where the R62W SNP seems to affect N-glycosylation by altering the tertiary or quaternary structure of the protein [45].

The expression of GPER P16L has been associated with increased blood pressure and accumulation of LDL-cholesterol in women [35, 46]. However, the role of polymorphic GPER in the cardiovascular system is apparently still controversial [47]. For breast cancer, one study associated this SNP with some histopathological features such as progesterone receptor status [48]. Thus, the clinical significance of the rs11544331 polymorphism remains to be clarified, in particular considering that the P16L variant is relatively common in the general population with an allelic frequency of about 20% [49]. Altogether, we have analyzed ten primary samples from breast cancer patients. One isolate of CAFs (CAFs_I) and one isolate of breast carcinoma cells (“epithelial cells”) from two different patients in Cosenza, Italy, proved to be homozygous for the polymorphism and homozygous wild-type, respectively. To our surprise, all eight samples of CAFs from patients in Geneva, Switzerland, turned out to be heterozygous for this polymorphism. The fact that their normal tissues were also heterozygous suggests that this specific SNP was there from the outset and did not arise as part of the tumorigenic selection. Our finding that nuclear forms of GPER regulate the transcription of cancer-relevant genes and may induce the secretion of factors by CAFs that regulate the migration of the carcinoma cells allows one to speculate that the P16L variant may be associated with a higher risk. However, given our small sample size, it would be premature to conclude that women with one or two alleles of this SNP indeed have an increased risk to develop breast cancer or to progress to a more aggressive disease. A much larger number of samples will be necessary to establish whether this SNP predisposes to breast cancer, and to which type of breast cancer.

### Functional repurposing of the polymorphic GPER variant P16L

The P16L polymorphism effectively converts GPER from a membrane-associated and signaling GPCR to a nuclear transcription factor-like molecule. Others had noticed that GPER P16L is defective for the activation of MAPK signaling in vascular smooth muscle cells of the rat aorta when stimulated with the synthetic GPER ligand G-1 [35]. While we confirmed that GPER P16L is not able to induce MAPK signaling when stimulated with OHT, we found that rather than being hypofunctional, GPER P16L relocalizes into the nucleus and acts as a transcription factor by binding to the regulatory sequences of the target genes *c-FOS* and *CTGF*. We know from our previous investigations that nuclear GPER, upon activation with an agonist such as E2 or OHT, induces proliferation and migration, most likely by regulating the expression of these and other target genes [16]. In the context of the paracrine effects of the CM from CAFs reported here, it remains to be seen what the contribution of IL1β is and whether its induction in CAFs [21] requires nuclear GPER.

Mechanistically speaking, how the repurposing of GPER to a transcription factor-like molecule really works must still be determined. Whereas other GPCRs may continue to signal inside the nucleus as GPCRs using most if not all components of the GPCR signaling machinery [25], others, such as F2rl1 and GPER may bind to chromatin directly or through tethering to a DNA-bound transcription factor. Obviously, this raises many more questions that will have to be addressed in the future. Our confocal microscopy images of nuclear GPER argue that it is diffusely present throughout the nucleoplasm rather than associated with the inner membrane of the nuclear envelope. If GPER is first targeted to a membrane upon synthesis before localizing to the nucleus, as suggested by the tunicamycin result, one wonders how this 7-transmembrane protein can be extracted from the membrane in a soluble form. If GPER is genuinely bifunctional, it should be elucidated how it binds chromatin and regulates transcription in an agonist-dependent fashion, and what partner proteins are involved. It will also be interesting to characterize its chromatin binding sites and target genes at a genome-wide level. This could provide further insights into the functional relevance of this alternate mode of action of GPER.

## MATERIALS AND METHODS

### Reagents

17β-estradiol (E2), 4-hydroxytamoxifen (OHT), and tunicamycin were purchased from Sigma-Aldrich. E2 and OHT were dissolved in ethanol, tunicamycin in dimethyl sulfoxide (DMSO). Primary antibodies were the following: against the FLAG tag (M2, Sigma Aldrich), α-tubulin (DM1A, Sigma Aldrich), histone H3 (ab1791, Abcam), pERK1/2 (E-4, Santa Cruz Biotech), ERK2 (C-14, Santa Cruz Biotech), GAPDH (ab9484, Abcam), ERα (HC-20, Santa Cruz Biotech), GPER (LS-A4272, LifeSpan Biosciences), vimentin (V9, Abcam), and E-cadherin (H-108, Santa Cruz Biotech).

### Generation of primary cells from breast cancer tissues

For primary samples from patients, signed informed consent from all the patients was obtained and all samples were collected, identified and used in accordance with protocols approved by the respective local ethical review committees.

All CAFs were extracted as previously described [10, 16, 17]. The original CAF isolate mentioned in these publications is referred to as CAFs_I to distinguish it from the new CAF isolates reported here; CAFs_I had been obtained from one patient who had undergone surgery at the Regional Hospital of Cosenza (Italy) and were maintained in culture in fibroblast growth medium [10, 16, 17]. Unrelated epithelial breast cancer cells were extracted from another patient with invasive mammary carcinoma who had undergone mastectomy at the Regional Hospital of Cosenza (Italy).

All new breast cancer specimens were collected from primary tumors of 5 patients with diagnosed invasive carcinomas (#1 to #5) and 3 patients with in-situ carcinomas (#6 to #8) who underwent surgery at the Cantonal University Hospital of Geneva (Switzerland). For each patient, a second population of fibroblasts was isolated from a noncancerous breast tissue obtained from the same breast.

To collect cells from primary tissue samples, the tissue was cut into smaller pieces (1-2 mm diameter) and incubated overnight at 37°C in digestion solution (400 IU collagenase, 100 IU hyaluronidase, and 10% fetal bovine serum [FBS] in Dulbecco's modified Eagle's medium [DMEM] containing antibiotics and anti-mycotic agents). The digested suspension was then centrifuged at 90 x g for 2 min; at this point, pellets and supernatants were enriched in epithelial and fibroblast cells, respectively. The epithelial cell pellets were resuspended and cultured in RPMI-1640 supplemented with 10% FBS. The supernatants containing the fibroblasts were centrifuged at 485 × g for 8 min; these pellets were then resuspended in fibroblast growth medium (Medium 199 and Ham's F12 mixed 1:1 and supplemented with 10% FBS) and cultured at 37°C in 5% CO_2_.

Primary cell cultures from breast specimens were characterized by immunofluorescence using vimentin and E-cadherin as mesenchymal and epithelial markers, respectively. Fibroblast activation was assessed by evaluating the mRNA expression of *FAP*, *ACTA2* and *CAV-1* by quantitative RT-PCR.

### Culture of cell lines

SkBr3 breast cancer cells were maintained in RPMI-1640 without phenol red, supplemented with 10% FBS. MDA-MB-231 and MCF-7 breast cancer cells were maintained in DMEM with phenol red, supplemented with 10% FBS.

### Plasmids and transfections

The following plasmids have been previously described: The short hairpin construct against human GPER (shGPER) and the shGPER-resistant version of the GPER expression plasmid (GPER Rescue) [12, 50]; plasmid 3x-FLAG-hGPER for expression of a FLAG-tagged version of GPER [51]; plasmid FLAG GPERΔNLS for expression of a FLAG-tagged NLS-defective GPER [16]. An expression vector for the N-glycosylation mutant GPER Rescue N25/32Q was generated by mutating two of the three codons for the N-glycosylation sites present in the N-terminal portion of human GPER. The mutagenesis was done by PCR with Phusion High Fidelity DNA Polymerase (New England Biolabs) and the following primers:5’-GCGGGTGGGACAGCTGG AGCTCGGGGGAGGTGGTCTGGGGGGCCGCAGG-3’ (forward primer) and5’-CCTGCGGCCCCCCAGACCACCTCCCCCGAGCTCCAGCTGTCCCACCCGC-3’ (reverse primer). The expression vectors for the P16L versions of FLAG-tagged GPER and GPER Rescue were generated by mutagenesis with the following primers: 5’-CTGCGCGGTGCCTAGGTACATCTCCAG-3’ (forward primer) and 5’-CTGGAGATGTACCTAGGCACCGCGCAG-3’ (reverse primer).

For knockdown experiments, SkBr3 cells were plated onto 10-cm dishes and transiently transfected in medium without serum for 24 hours with a control shRNA or the shGPER. For the experiments performed using GPER Rescue, SkBr3 cells were transfected for 24 hours, in serum-free medium, with shGPER and then co-transfected for additional 24 hours with GPER Rescue. For the expression of 3xFLAG GPER or FLAG GPERΔNLS, SkBr3 cells were seeded onto 10-cm dishes and transfected with empty vector or expression plasmids for 24 hours in serum-free medium. All the transient transfections in SkBr3 were performed using polyethylenimine as transfection reagent. Transfection experiments carried out with CAFs were performed in serum-free DMEM with Lipofectamine 3000 (Life Technologies) as transfection reagent.

### Cell lysates, cell fractionation and immunoblotting

To obtain total cell lysates SkBr3 cells, cells from a 10-cm dish were lysed in 500 μl of 50 mM Tris-HCl pH 8, 50 mM NaCl, 1.5 mM MgCl2, 1 mM EGTA, 10% glycerol, 1% Triton X-100, 1% SDS and protease inhibitor cocktail. Cells extracted from breast cancer biopsies were grown in normal growth medium and lysed using RIPA Buffer (50 mM Tris-HCl pH 7.4, 1% NP40, 0.5% sodium deoxycholate, 0.1% SDS, 2 mM EDTA and protease inhibitor cocktail).

For cell fractionation experiments, SkBr3 cells were grown in 10-cm dishes in regular growth medium and starved of serum for 24 hours before transfection. Cells were then washed twice with ice-cold phosphate-buffered saline (PBS), scraped from the plate and lysed in 500 μl of subcellular fractionation buffer (SF) (250 mM sucrose, 20 mM HEPES pH 7.4, 10 mM KCl, 1,5 mM MgCl_2_, 1mM EDTA, 1 mM EGTA, 1 mM DTT, and protease inhibitor cocktail) by agitation for 30 min at 4 °C. This was followed by centrifugation at 720 x g for 5 min at 4 °C. The supernatant was removed and transferred to a new tube; after centrifugation at 10000 x g for 10 min at 4 °C, this supernatant was considered the cytosolic fraction. The pellet of the original cell lysates was washed twice with SF and centrifuged at 720 x g for 10 min at 4 °C. The pellet, containing the nuclei, was resuspended in nuclear lysis buffer (50 mM Tris-HCl pH 8, 150 mM NaCl, 1% NP40, 0.5% sodium deoxycholate, 0.1% SDS, 10% glycerol and protease inhibitor cocktail), incubated at 4 °C for 15 min before sonication for 3 times 3 seconds at 30 % of full amplitude power using the Bioruptor sonicator (Diagenode). This was considered the nuclear fraction.

For immunoglotting, nitrocellulose membranes were probed overnight at 4°C with the appropriate primary antibodies. The secondary antibodies were IRDye 680LT and IRDye 800 CW for quantitation with the Odyssey infrared imaging system (LI-COR).

### Immunofluorescence and confocal microscopy

Cells grown on glass coverslips were fixed with 4% paraformaldehyde in PBS for 15 min at room temperature, washed with PBS, permeabilized with 0.1% Tween-20 three times for 5 min and then blocked for 30 min at room temperature with PBS containing 10% FBS, 0.1% Triton X-100, and 0.05% Tween-20. Incubation with primary antibodies was overnight at 4°C, with secondary antibodies (fluorescein-conjugated anti-rabbit IgG or Texas red-conjugated anti-mouse IgG antibodies from Vector Laboratories) for 1 hour at room temperature. Cells were imaged with a Zeiss LSM 700 laser scanning confocal microscope.

### Direct sequencing of PCR amplicons

Genomic DNA was subjected to PCR in order to generate specific *GPER1* amplicons of 411 bp with the following primers: 5’-GATGTGACTTCCCAAGCCCG-3’ (forward primer); 5’-CTGCAGGAAGAGCGACATGA-3’ (reverse primer). PCR products were purified with the Invisorb fragment cleanup kit (Stratec Biomedical) and the relevant region sequenced directly using the following reverse primer: 5’-GAGAGGAACAGGCCGATCAC-3’.

### GPER SNP analysis

GPER missense SNP rs11544331 [48] leading to the amino acid substitution P16L was revealed in genomic DNA-containing lysates with a tetra-primer PCR analysis protocol allowing allele-specific amplification [33] using the following primers: 5’-AACAAACCCAACCCAAACCACCACAGGT-3’ (Outer Forward Primer), 5’-AGCCGATGGGGAAGAGGAAGATGGTGTA-3’ (Outer Reverse Primer), 5’-GGGCGTGGGCCTGGAGATGTAACC-3’ (Inner-C Primer), 5’-CGCAGGCTGCGCGGTGCATA-3’ (Inner-T Primer). PCR conditions were: 95 °C for 5 min, 35 cycles of 95 °C for 30 seconds, 60 °C for 90 seconds and 72 °C for 30 seconds, final extension at 68 °C for 10 min. PCR products were analyzed by electrophoresis on 2% agarose gels. To further confirm the presence of the SNP rs11544331, the purified amplicons, used for DNA sequencing, were subjected to enzymatic digestion using the restriction enzyme AvrII and separated on a 1.5% agarose gel.

### Chromatin immunoprecipitation

ChIP experiments were performed as previously described [52]. Where indicated, cells were treated with tunicamycin (or the vehicle DMSO) in serum-free medium for 8 hours. Regions containing AT-rich sequences located within the promoters of the *c-FOS* (-46 to -41) [53] or *CTGF* (from -377 to -142) [16] genes, and a negative control sequence were amplified with the following primer pairs: 5’-AGGGAGCTGCGAGCGCTGGG-3’ (forward primer) and 5’-GTGGCGGTTAGGAGGCAAAGCCG-3’ (reverse primer) for c-FOS, 5’-GCTTTTTCAGACGGAGGAAT-3’ (forward primer) and 5’-GAGCTGGAGGGTGGAGTCGC-3’ (reverse primer) for *CTGF*, 5’-TGGCCCTTGATACTGGAGTC-3’ and 5’-GACATCCAAGGCAAGATGGT-3’ for the negative control region. The gene-specific values were normalized to their corresponding inputs and the internal standard (control region) and represented as the fold enrichment relative to DMSO-treated cells.

### Reverse transcription and quantitative RT-PCR

Total RNA was extracted with guanidinium-acid-phenol as previously described [54]. After reverse transcription with the random primer hexadeoxynucleotide mix (Promega), quantitation was done by real-time PCR with the following primers: 5’-AGAAGAAAGCAGAAC TGGATGG-3’ (forward primer) and 5’-ACACACTTCTTGCTTGGAGGAT-3’ (reverse primer) for *FAP*, 5’-AGGAAGGACCTCTATGCTAACAAT-3’ (forward primer) and 5’-AACACATAGGTAACGAGTCAGAGC-3’ (reverse primer) for *ACTA2*, 5’-GTCAACCGCGAC CCTAAACA-3’ (forward primer) and 5’-GAAGCTGGCCTTCCAAATGC-3’ (reverse primer) for *CAV1*, 5’-GCTCTTGGACAGGAACCAG-3’ (forward primer) and 5’-AAGATCTCCACCATGCCCTCT-3’ (reverse primer) for *ESR1*, 5’- GATGTGACTTCCCAAGCCCG-3’ (forward primer) and 5’-GAGAGGAACAGGCCGATCAC-3’ (reverse primer) for *GPER1*, and 5’-GCACAACAGGAAGAGAGAGACC-3’ (forward primer) and 5’-AGGGGAGATTCAGTGTGGTG-3’(reverse primer) for *GAPDH*.

### Cell migration assays

CAFs were cultured in regular growth medium to 80% confluence. Then, cells were washed twice with PBS and transfected in serum-free DMEM with empty vector, shGPER or FLAG GPERP16L using Lipofectamine 3000 (Life Technologies) for 24 hours. Subsequently, cells were treated with 100 nM 17β-estradiol for additional 24 hours. Thereafter, the culture supernatant was collected, centrifuged at 16’000 g for 5 min to remove cell debris, filtered through a 0.45 μm filter and used as CM in migration assays with MDA-MB-231 cells. Boyden chamber assays were done with Transwell 8 μm polycarbonate membrane inserts (Costar). 2 × 10^5^ cells were inoculated in serum-free medium in the upper chamber and 750 μl of CM was added into the lower chamber as a chemoattractant. After 18 hours of incubation, cells were fixed with formaldehyde and permeabilized with methanol. MDA-MB-231 cells on the upper surface of the membrane were removed by wiping with a cotton swab, whereas the migrated cells on the side facing in the lower chamber were stained with 0.5% crystal violet. Migrated cells were counted in ten randomized fields photographed with a Dino-Lite ocular camera.

### Statistical analysis

Two sided non-paired Student's t-tests were done to determine differences between two groups.

## SUPPLEMENTARY MATERIALS FIGURES AND TABLES


