# Supplementary material for: A genetic polymorphism repurposes the G-protein coupled and membrane-associated estrogen receptor GPER to a transcription factor-like molecule promoting paracrine signaling between stroma and breast carcinoma cells
Source: Oncotarget. 2017 May 24;8(29):46728–44. doi: 10.18632/oncotarget.18156 (PMC5564519; doi:10.18632/oncotarget.18156)
Supplement: Supplementary file 1 [file oncotarget-08-46728-s001.pdf]

A genetic polymorphism repurposes the G-protein coupled and membrane-associated estrogen receptor GPER to a transcription factor-like molecule promoting paracrine signaling between stroma and breast carcinoma cells

Supplementary Materials

A

NetNGlyc 1.0 Server - prediction results

Technical University of Denmark

```
   Asn-Xaa-Ser/Thr sequons in the sequence output below are highlighted in blue.
        Asparagines predicted to be N-glycosylated are highlighted in red.
```

Output for 'Sequence'

```
################################################################################

    Warning: This sequence may not contain a signal peptide!:

    Proteins without signal peptides are unlikely to be exposed to
    the N-glycosylation machinery and thus may not be glycosylated
    (in vivo) even though they contain potential motifs.

    SignalP-NN euk predictions are as follows:

  # name                    Cmax  pos ?  Ymax  pos ?  Smax  pos ?  Smean ?  D      ?
  Sequence                     0.122  22  0.112  30  0.194  19  0.099   0.107 N  0.500      SignalP-TM

    SignalP output is explained at http://www.cbs.dtu.dk/services/SignalP/output.html

################################################################################

Name:  Sequence         Length:  375
MDVTSQARGVGLEMYPGTAQPAAPNTTSPELNLSHPLLGTALANGTGELSEHQQYVIGLFLSCLYTIFLFPIGFVGNILI      80
LVVNISFREKMTIPDLYFINLAVADLILVADSLIEVFNLHERYYDIAVLCTFMSLFLQVNMYSSVFFLTWMSFDRYIALA     160
RAMRCSLFRTKHHARLSCGLIWMASVSATLVPFTAVHLQHTDEACFCFADVREVQWLEVTLGFIVPFAIIGLCYSLIVRV     240
LVRAHRHRGLRPRRQKALRMILAVVLVFFVCWLPENVFISVHLLQRTQPGAAPCKQSFRHAHPLTGHIVNLAAFSNSCLN     320
PLIYSFLGETFRDKLRLYIEQKTNLPALNRFCHAALKAVIPDSTEQSDVRFSSAV
........................N......N...........N....................................      80
...N............................................................................     160
................................................................................     240
................................................................................     320
.......................................................                              400

(Threshold=0.5)
----------------------------------------------------------------------
SeqName      Position  Potential   Jury     N-Glyc
                                 agreement  result
----------------------------------------------------------------------
Sequence        25 NTTS   0.7813     (9/9)    +++
Sequence        32 NLSH   0.6642     (9/9)    ++
Sequence        44 NGTG   0.7072     (9/9)    ++
Sequence        84 NISF   0.5674     (8/9)    +
```

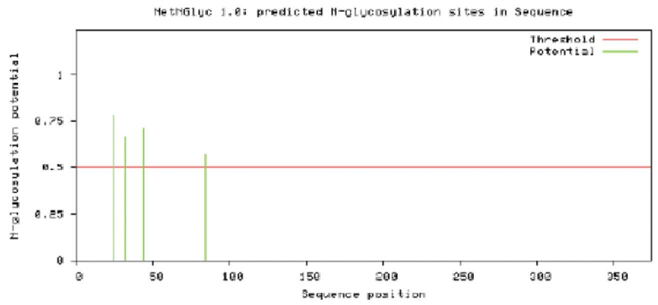

Graphics in PostScript

Explain the output. Go back.

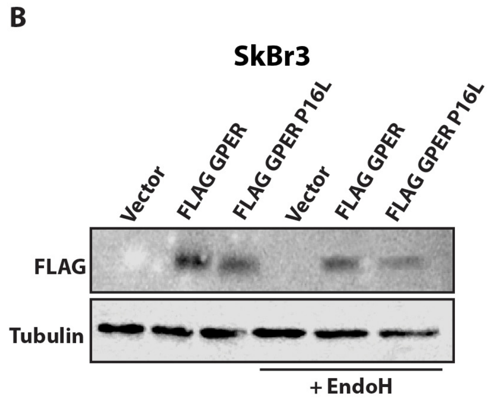

Supplementary Figure 1: A. Screenshot of the results generated by the NetNGlyc 1.0 online prediction tool. It indicates three high confidence N-linked glycosylation sites in the N-terminal portion of GPER. **B**. Immunoblots of extracts from SkBr3 breast cancer cells transfected wild-type and mutant FLAG-tagged GPER as indicated. Where indicated, extracts were treated for 18 hours with the enzyme EndoH. Tubulin was used as loading control. The image shown is representative of two independent experiments.

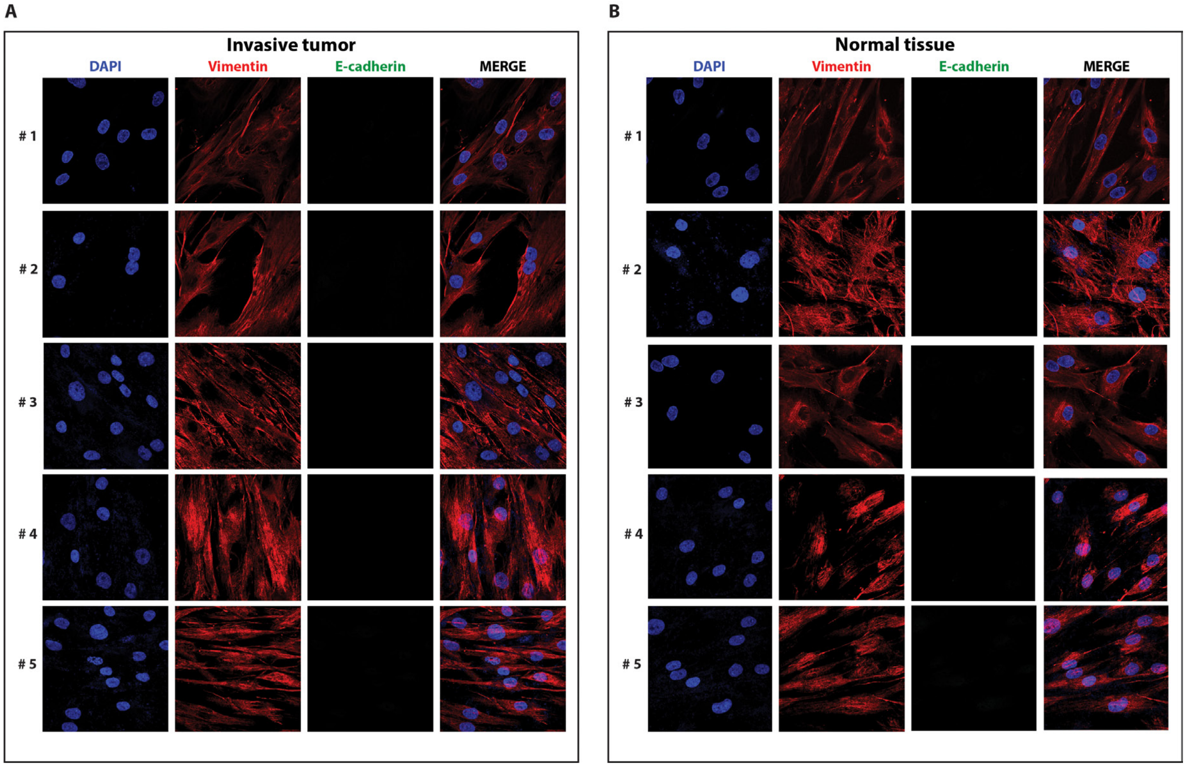

Supplementary Figure 2: Fibroblast cells obtained from patients affected by invasive breast tumors express vimentin but not E-cadherin. Representative immunofluorescence micrographs of cells obtained from invasive tumor biopsies (**A**.) and from the corresponding normal tissues (**B**.) of patients #1 to #5. Cells were immunostained with anti-vimentin (red) as well as anti-E-cadherin (green) antibodies and analyzed by confocal microscopy. Nuclei (in blue) were stained using DAPI. Images shown are representative of 10 different random fields.

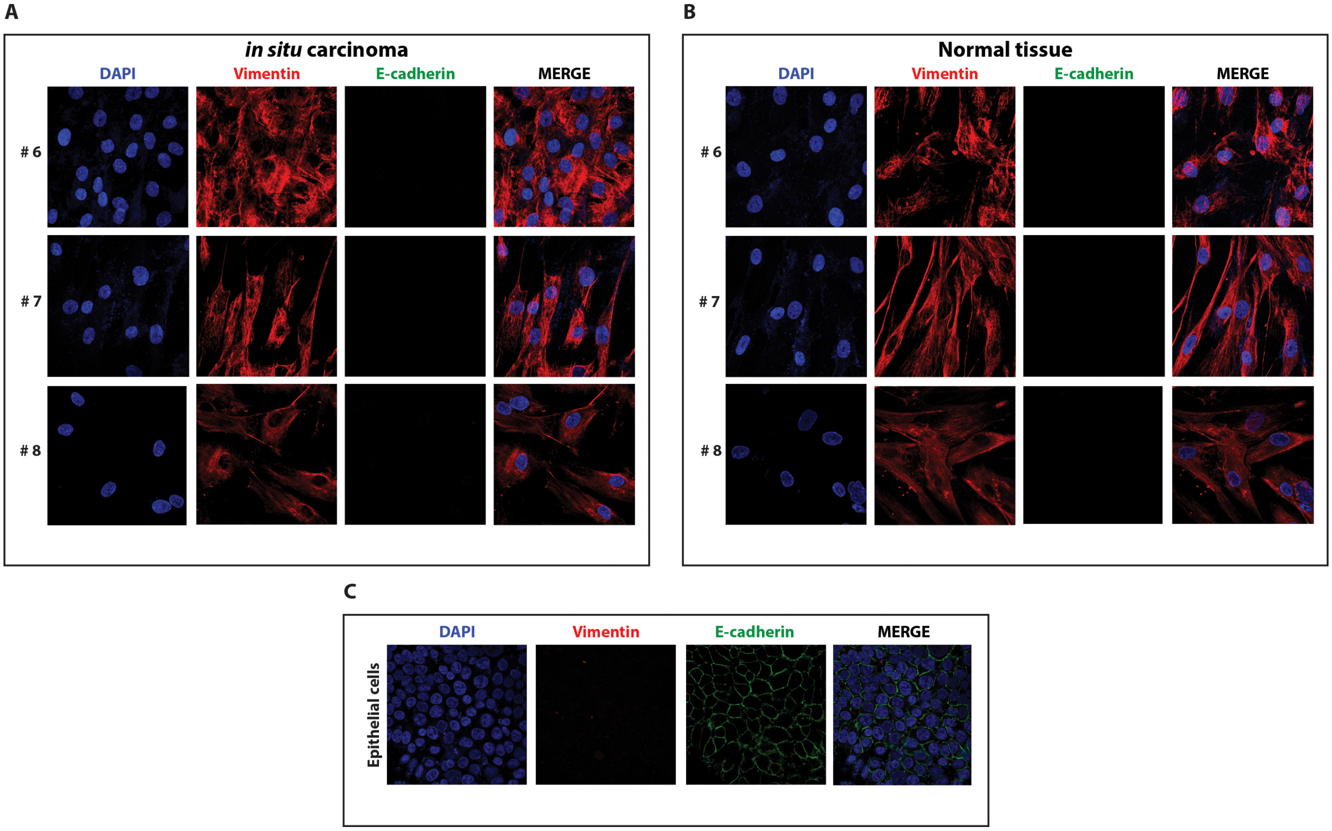

Supplementary Figure 3: Fibroblast cells obtained from patients affected by in-situ breast carcinoma express vimentin but not E-cadherin. Representative immunofluorescence micrographs of cells obtained from invasive tumor biopsies (**A**.) and from the corresponding normal tissues (**B**.) of patients #6 to #8. **C**. Representative images of epithelial cells obtained from an invasive breast cancer and used as positive control for E-cadherin expression.

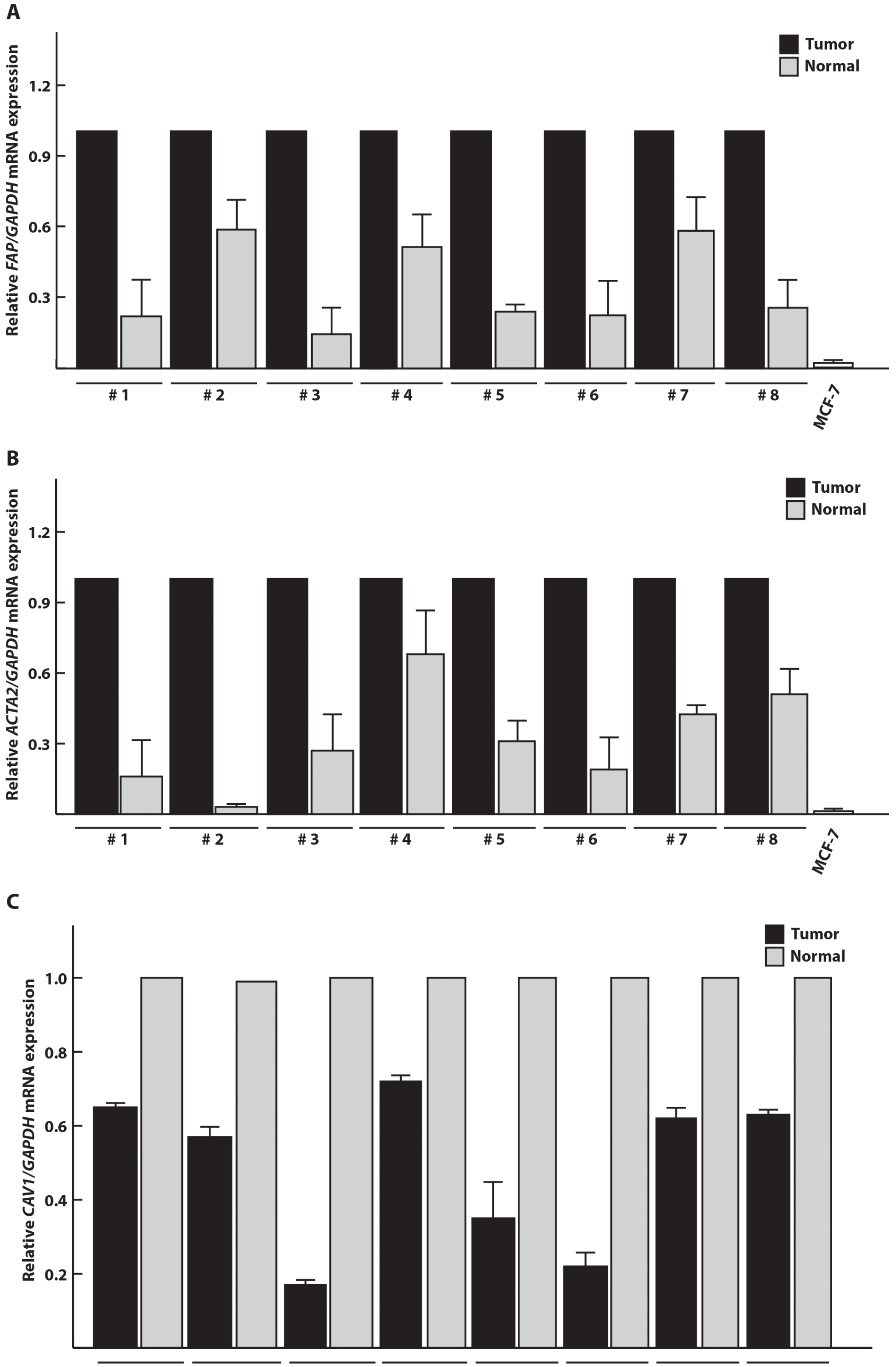

Supplementary Figure 4: Fibroblasts obtained from invasive and in-situ breast carcinomas express the CAF markers. The mRNA levels for the markers *FAP* (**A.**), *ACTA2* (**B.**) and *CAV1* (**C.**) were measured by quantitative RT-PCR and standardized to *GAPDH*. Values are presented as fold changes relative to the tumor or normal samples (mean ± SD) and represent three independent experiments with triplicate samples.

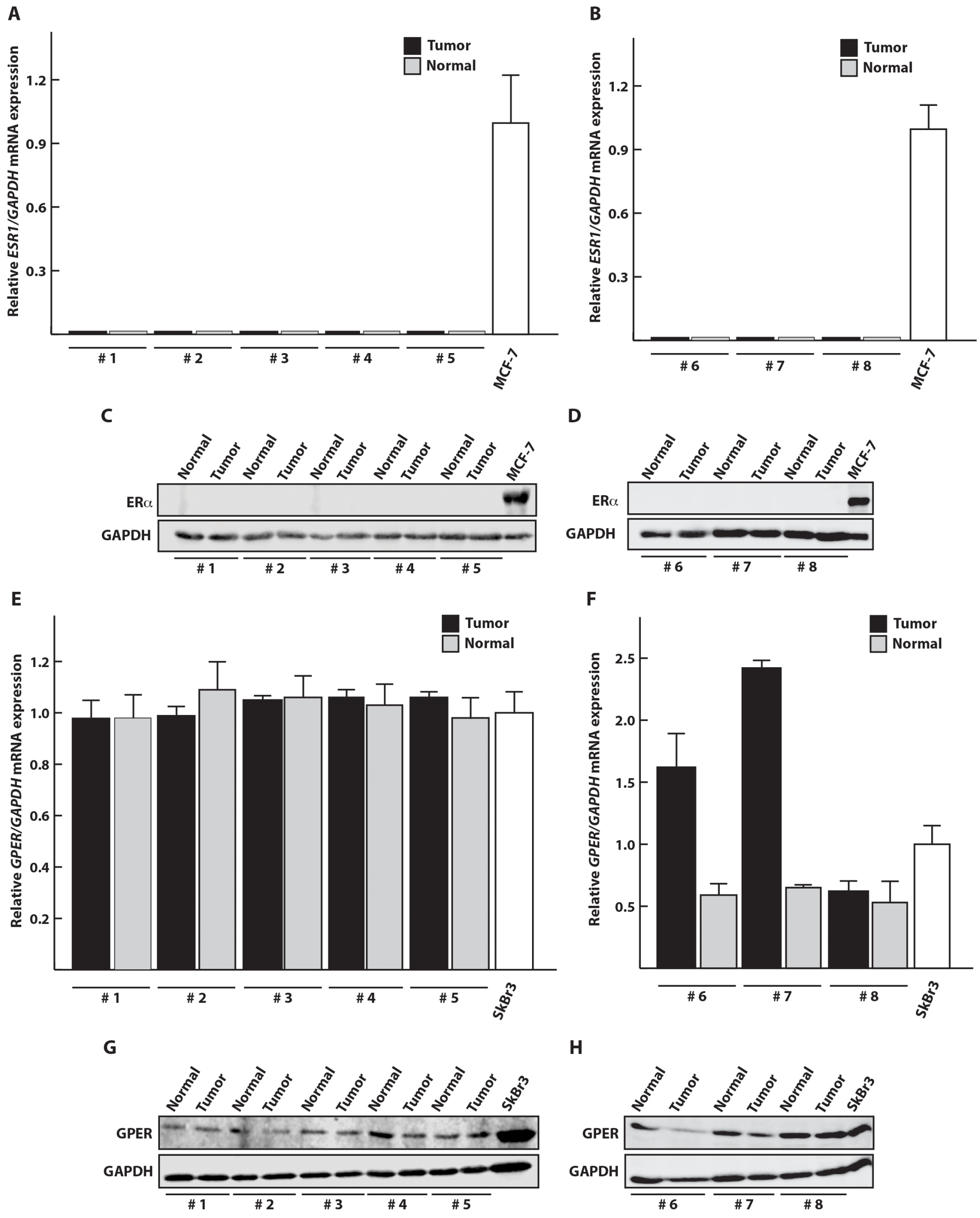

Supplementary Figure 5: A. and **B**. Evaluation of *ESR1* mRNA levels in isolated fibroblasts by quantitative RT- PCR. Values were normalized to *GAPDHI* and expressed as fold changes (mean ± SD) relative to the *ESR1* mRNA levels in MCF-7 breast cancer cells. **C**. and **D**. Immunoblots showing ERα expression in fibroblasts compared to MCF-7 cells. **E**./**F**. and **G**./**H**. show the corresponding analyses for *GPER* mRNA and GPER protein levels, respectively. In this case, SkBR3 cells were used as positive control. Patients #1-5 and #6-8 had invasive and in-situ carcinoma, respectively.

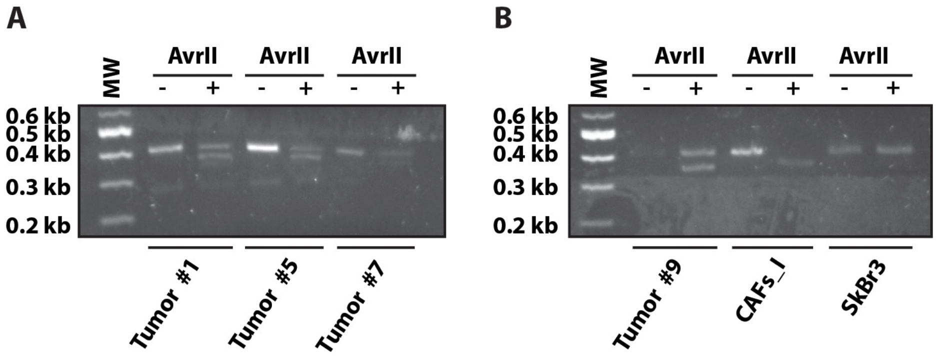

Supplementary Figure 6: CAFs from the new set of samples are heterozygous for the polymorphism P16L. **A**. and **B**. Agarose gel electrophoresis of *GPER* amplicons digested with AvrII where indicated (CAFs from patients #1, #5, #7, and #9, and for comparison, CAFs_I and SkBr3 cells). The bands above and below 400 bp indicate the presence of the C and T alleles, respectively.

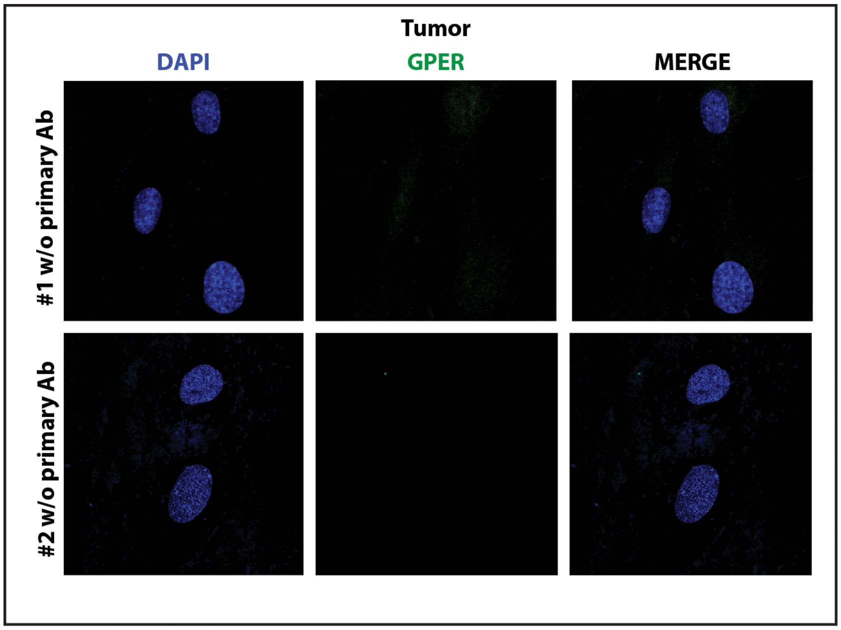

Supplementary Figure 7: Immunofluorescence control experiments. Representative images obtained with CAFs from two invasive breast cancer biopsies of patients #1 and #2, immunostained only with the secondary antibody.
